# Sevoflurane Exposure Induces Neuronal Cell Parthanatos Initiated by DNA Damage in the Developing Brain via an Increase of Intracellular Reactive Oxygen Species

**DOI:** 10.3389/fncel.2020.583782

**Published:** 2020-12-03

**Authors:** Meihua Piao, Yingying Wang, Nan Liu, Xuedong Wang, Rui Chen, Jing Qin, Pengfei Ge, Chunsheng Feng

**Affiliations:** ^1^Department of Anesthesiology, The First Hospital of Jilin University, Changchun, China; ^2^Department of Neurosurgery, The First Hospital of Jilin University, Changchun, China

**Keywords:** sevoflurane, Parthanatos, DNA damage, oxidative stress, developing brain, neurotoxicity

## Abstract

The safety of volatile anesthetics in infants and young children has been drawing increasing concern due to its potential neurotoxicity in the developing brain. Neuronal death is considered a major factor associated with developmental neurotoxicity after exposure to volatile anesthetics sevoflurane, but its mechanism remains elusive. Parthanatos, a new type of programmed cell death, resulting from poly (ADP-ribose) polymerase 1 (PARP-1) hyperactivation in response to DNA damage, was found to account for the pathogenesis of multiple neurological disorders. However, the role of Parthanatos in sevoflurane-induced neonatal neuronal cell death has not been investigated. To test it, neuronal cells treated with 2, 4, and 8% sevoflurane for 6, 12, and 24 h and postnatal day 7 rats exposed to 2.5% sevoflurane for 6 h were used in the present study. Our results found sevoflurane exposure induced neuronal cell death, which was accompanied by PARP-1 hyperactivation, cytoplasmic polymerized ADP-ribose (PAR) accumulation, mitochondrial depolarization, and apoptosis-inducing factor (AIF) nuclear translocation in the neuronal cells and hippocampi of rats. Pharmacological or genetic inhibition of PAPR-1 significantly alleviated sevoflurane-induced neuronal cell death and accumulation of PAR polymer and AIF nuclear translocation, which were consistent with the features of Parthanatos. We observed *in vitro* and *in vivo* that sevoflurane exposure resulted in DNA damage, given that 8-hydroxydeoxyguanosine (8-OHdG) and phosphorylation of histone variant H2AX (γH2AX) were improved. Moreover, we detected that sevoflurane exposure was associated with an overproduction of intracellular reactive oxygen species (ROS). Inhibition of ROS with antioxidant NAC markedly alleviated DNA damage caused by sevoflurane, indicating that ROS participated in the regulation of sevoflurane-induced DNA damage. Additionally, sevoflurane exposure resulted in upregulation of Parthanatos-related proteins and neuronal cell death, which were significantly attenuated by pretreatment with NAC. Therefore, these results suggest that sevoflurane exposure induces neuronal cell Parthanatos initiated by DNA damage in the developing brain via the increase of intracellular ROS.

## Introduction

The safety of volatile anesthetics in infants and young children has been drawing increasing concern due to its potential neurotoxicity in the developing brain. Extensive studies have documented that the volatile anesthetic, sevoflurane, induced widespread neurodegeneration and long-term cognitive dysfunction in neonatal rodents and non-human primates (Gentry et al., 2013; Zheng et al., 2013; Amrock et al., 2015; Liu et al., 2015; Alvarado et al., 2017; Perez-Zoghbi et al., 2017), which is particularly evident for prolonged or repeated exposure. Emerging data demonstrated in clinical practice that exposure to volatile anesthetics in the early period of infants was linked with abnormal neurobehavioral changes in childhood (Diaz et al., 2016; Ing et al., 2017). Although accumulating evidence proved that neuronal cell death was considered a major factor associated with volatile anesthetics-induced developmental neurotoxicity, and volatile anesthetics were found to trigger neuronal cell death via induction of apoptosis, autophagy, and ferroptosis in the developing brain (Zheng et al., 2013; Liu et al., 2015; Olutoye et al., 2016; Xia et al., 2018; Wang et al., 2019), the underlying mechanisms of neonatal neuronal cell death following sevoflurane exposure remain poorly understood.

Parthanatos, a newly recognized modality of programmed cell death that occurs as a result of poly (ADP-ribose) polymerase 1 (PARP-1) hyperactivation, is characterized by excessive accumulation of cytoplasmic polymerized ADP-ribose (PAR), which resulted in the decline of mitochondrial membrane potential and nuclear translocation of apoptosis-inducing factor (AIF), leading to chromatin condensation and cell death (Yu et al., 2002, 2006; Wang et al., 2009; Fatokun et al., 2014). Distinguished from other types of cell death, Parthanatos neither results in cell swelling nor induces the formation of apoptotic bodies and autophagosome (Fatokun et al., 2014). Increasing evidence has implicated Parthanatos in the pathogenesis of various neurological diseases including brain trauma, stroke, Alzheimer’s Disease, and Parkinson’s Disease (Pacher and Szabo, 2008; Lee et al., 2013, 2014; Martire et al., 2015; Conrad et al., 2016; Kam et al., 2018). However, it is largely unknown whether Parthanatos participates in neuronal cell death induced by sevoflurane exposure in the developing brain, and additionally, whether inhibition of Parthanatos would provide a potential strategy to prevent sevoflurane-induced developmental neurotoxicity.

PARP-1, a highly expressed nuclear enzyme, is well acknowledged as a DNA damage sensor, which constitutes a DNA base-excision repair system by sensing DNA strand nicks and breaks, and functions to facilitate multiple facets of DNA damage repair and cell survival through the synthesis of PAR polymer by consuming NAD and ATP (Kim et al., 2005; Khodyreva et al., 2010; Wang et al., 2012, 2018; Fatokun et al., 2014). Whereas upon extensive DNA damage, PARP-1 is overactivated followed by excess PAR polymer accumulation and AIF release into the nucleus, leading to the occurrence of Parthanatos (Fatokun et al., 2014). Although genomic DNA was attacked by various genotoxic agents, growing evidence supported that oxidative stress induced by intracellular reactive oxygen species (ROS) played a crucial role in regulating DNA damage which initiated PARP-1-dependent cell death response (Finkel and Holbrook, 2000; Chiu et al., 2011; Wang et al., 2018). In addition, several studies reported that sevoflurane exposure-induced neuronal cell death was associated with the accumulation of intracellular ROS, mitochondrial depolarization, and ATP depletion in neonatal rodents (Liu et al., 2015; Xu et al., 2017). Evidence from clinical data also provided that exposure of sevoflurane resulted in exacerbated DNA damage and oxidative status (Baysal et al., 2009; Costa Paes et al., 2014). Given the above, sevoflurane-induced neuronal death was accompanied with oxidative stress which linked the DNA damage to cell death triggered by PARP-1 overactivation. Thus, we speculate that sevoflurane results in overproduction of ROS, which causes DNA damage, thereby causing excessive PARP-1 activation and AIF nuclear translocation, subsequently leading to neuronal cell Parthanatos in the developing brain.

Therefore, in the present study, we aim to investigate whether Parthanatos is involved in neuronal cell death following sevoflurane exposure using human SH-SY5Y neuroblastoma cells, mouse hippocampal HT22 cells, primary rat hippocampal neurons, and postnatal day 7 (P7) rats, then reveal the potential role of oxidative stress-initiated DNA damage in determining the mechanisms of sevoflurane-triggered Parthanatos in the developing brain.

## Materials and Methods

### Antibodies and Reagents

Sevoflurane was purchased from Maruishi Pharmaceutical Co. (Osaka, Japan). *N*-acetyl-L-cysteine (NAC), 3-aminobenzamide (3AB), and hydrogen peroxide (H_2_O_2_) were purchased from Sigma-Aldrich Company (St. Louis, MO, United States). Lactate dehydrogenase (LDH) detection kit was from Beyotime Biotech (Nanjing, China). Nuclear and Cytoplasmic Protein Extraction Kit (P0028) and BCA Protein Assay Kit (P0010) were purchased from Beyotime Biotech (Shanghai, China). Anti-PAR polymer (AM80) was from Calbiochem company (Danvers, MA, United States). Anti-PARP-1 (ab32138), anti-AIF (ab32516), and anti-γH2AX (ab26350) were purchased from Abcam Company (Cambridge, MA, United States). Anti-ATM (sc-135663) and anti-p-ATM (sc-47739) were from Santa Cruz Biotechnology (Santa Cruz, CA, United States). Anti-8-OHdG (251640) was from Abbiotec (San Diego, CA, United States). PARP-1 small interfering RNA (SiRNA) was purchased from GenePharma Company (Suzhou, China). Anti-Histone-H3 (17168-1-AP) was from Proteintech Group. Anti-β-actin (AA128) was from Beyotime Biotech (Nanjing, China).

### Animals

All animal procedures were consistent with the National Institutes of Health Guidelines for the Care and Use of Laboratory Animals and approved by the Ethics Committee of The First Hospital of Jilin University. Postnatal day 7 (P7) Sprague-Dawley (SD) rat pups were supplied by the Yisi Institute of Laboratory Animal Technology (Changchun, China). Pups were group housed under standard laboratory conditions (12/12-h light/dark cycle, 22 ± 2°C, 60% humidity) with free access to food and water and allowed to stay in cages with maternal rats until sacrifice. All efforts were taken to minimize the number of animals used and their suffering.

### Cell Cultures

Human SH-SY5Y neuroblastoma cells and mouse hippocampal HT22 cells obtained from Shanghai Institute of Cell Biology, Chinese Academy of Sciences (Shanghai, China), were cultured in DMEM supplemented with 10% fetal bovine serum (FBS). The harvest of rat primary hippocampal neurons from newborn SD rats (within 24 h) was performed as described previously (Li et al., 2015). Hippocampal tissues were digested by trypsinization and terminated by adding DMEM with 10% FBS before being seeded onto a poly-D-lysine-coated 6 or 24-well culture plate. After being cultured with DMEM for 4 h, cells were maintained in Neurobasal Medium (Gibco, United States) supplemented with 2% B_27_, 0.5 mM L-Glutamine, 1 mM sodium pyruvate, 100 U/ml penicillin, and 100 μg/ml streptomycin. Hippocampal neurons were used for experiments at 7 days *in vitro*. All cells were cultured at 37°C in a humidified environment (5% CO_2_, 95% air).

### Sevoflurane Exposure and Treatment

For *in vitro* studies, all cells received with or without sevoflurane in a gas mixture of 5% CO_2_, 21% O_2_, and balanced N_2_ at 37°C in a tightly sealed plastic chamber (Billups-Rothenberg, Del Mar, United States) inside a cell culture incubator. The humidified gas mixture went through an agent-specific vaporizer at a flow rate of 2 L/min for 5 min and 0.5 L/min for the remaining exposure time. The cells were treated with sevoflurane at concentrations of 0, 2, 4, and 8% for 6, 12, and 24 h. The effluent gas of sevoflurane, O_2_, and CO_2_ in the chamber was monitored and maintained at a designed concentration throughout the experiment using an infrared monitor (Ohmeda 5330, Datex-Ohmeda, Louisville, Co.). To detect the role of PARP-1 and ROS in sevoflurane-induced neuronal cell death, cells exposed to sevoflurane at indicated concentrations for 12 h were pretreated in the presence or absence of PARP-1 inhibitor 3AB (500 μmol/L) or antioxidant NAC (5 mmol/L) at 1 h prior to sevoflurane exposure or H_2_O_2_ (250 μmol/L) incubation.

For *in vivo* studies, one hundred and eighty P7 rat pups from twenty litters (each litter has 8–12 pups) including both male and female were randomly divided into six groups (*n* = 30 per group): control group, sevoflurane group, 3AB group, sevoflurane+3AB group, NAC group, and sevoflurane+NAC group. Pups were administered with 3AB (30 mg/kg) or NAC (90 mg/kg) intraperitoneally (i.p.) in the same volume of approximately 0.2 ml at 1 h before the onset of sevoflurane exposure. Pups were placed in a gas-tight transparent anesthesia chamber with sevoflurane passing through a calibrated vaporizer. Anesthesia was induced with 5% sevoflurane in 60% O_2_ until the loss of righting reflex and maintained with 2.5% sevoflurane in 60% O_2_ at a flow rate of 2 L/min for 6 h. The pups without sevoflurane anesthesia were only exposed to oxygen at the same concentration and flux in an identical chamber. Gas phase concentrations in the chamber was continuously monitored as the *in vitro* studies were performed. Sodium lime existed in the bottom of the chamber to absorb CO_2_. Rectal temperature was continuously monitored to keep normothermic at 37 ± 0.5°C by using a heating pad under the chamber throughout the experiment. To avoid respiratory inhibition during sevoflurane anesthesia, pups were removed from the chamber every 2 h and provided stimulation with massage (Amrock et al., 2015). At the end of anesthesia, separate pups of six per group were euthanized with 5% isoflurane in 60% oxygen (Perez-Zoghbi et al., 2017) and immediate thoracotomy was performed to withdraw arterial blood samples from the left cardiac ventricle for blood gas analysis using a GEM Premier 3000 blood gas analyzer (Instrumentation Laboratory Co., United States). The other pups were recovered with 60% oxygen at a flow rate of 3 L/min until return of the righting reflex, then the pups were brought back to their maternal rats until sacrifice. The mortality rate of rat pups following anesthesia with 2.5% sevoflurane for 6 h in the present study was about 10%.

### Brain Tissue Preparation

After the rat pups were euthanized with 5% isoflurane as mentioned above, brain tissues were harvested as we previously described (Feng et al., 2016). In brief, for western blotting analyses and enzyme-linked immunosorbent assay (ELISA), pups of twelve per group at 6 h after sevoflurane exposure were transcardially perfused with ice cold phosphate buffered saline (PBS), and the brains were collected immediately to acquire the hippocampus. For HE staining, pups of six per group at 7 days after sevoflurane exposure were fixed with 4% paraformaldehyde following ice cold PBS transcardial perfusion, and then the brains were removed and post-fixed. All rat pups were perfused by an experienced experimenter.

### Cell Viability Assay

The SH-SY5Y (1 × 10^4^ cells/well) and HT22 (1 × 10^4^ cells/well) cells were seeded in a 96-well cell culture plate for 24 h, and the primary hippocampal neurons (2 × 10^5^ cells/well) were seeded in a 24-well cell culture plate for 7 days. All cells were treated with or without sevoflurane at indicated concentrations in the presence or absence of 3AB or NAC. According to manufacturer’s protocol, the cell viability was examined by measuring the levels of formazan using methyl thiazolyl tetrazolium (MTT) (Sigma-Aldrich, St. Louis, MO, United States) through reading the absorbance value at 490 nm (HTS 7000, Perkin Elmer, Boston, MA, United States).

### LDH Release Assay

Cells were cultured and treated as in the MTT assay. Lactate dehydrogenase (LDH) release assay was performed as we previously described (Wang et al., 2018). According to manufacturer’s protocol, cell death rate was examined by measuring the levels of LDH using a detection kit (Beyotime Biotech, Nanjing, China) through reading the absorbance value at 490 nm (HTS 7000, Perkin Elmer, Boston, MA, United States).

### Comet Assay

Comet assay, also called single cell gel electrophoresis (SCGE) assay, was performed as we previously described (Zhou et al., 2017). The SH-SY5Y cells (5 × 10^5^ cells/ml) and HT22 cells (5 × 10^5^ cells/ml) were seeded in a 6-well culture plate for 24 h and then exposed to sevoflurane at concentrations of 4 and 8% for 12 h in the presence or absence of NAC. Briefly, 100 μl cell suspension mixed with low-melting agarose were deposited on 1% agarose-prelayered slides. After solidification, the slides were immersed into the lysing solution at 4°C in dark for 1.5 h. Then the slides were electrophoresed in an electrophoresis tray filled with TBE buffer for 25 min. For alkaline comet assay, slides were placed in alkaline solution for 1 h and electrophoresed for 25 min. After being neutralized with 0.4 M Tris, cells were stained with ethidium bromide (EB) for 20 min in darkness. After being washed with PBS buffer three times, the slides were observed under fluorescence microscope (Olympus IX71, Tokyo, Japan).

### Intracellular ROS Analysis

The SH-SY5Y (1 × 10^4^ cells/well) and HT22 (1 × 10^4^ cells/well) cells were seeded in a 96-well cell culture plate for 24 h, and the primary hippocampal neurons (2 × 10^5^ cells/well) were seeded in a 24-well cell culture plate for 7 days. All cells were exposed to sevoflurane at concentrations of 4 and 8% for 12 h in the presence or absence of NAC. The levels of ROS were evaluated by redox-sensitive dye dichloro-dihydro-fluorescein diacetate (DCFH-DA) (Beyotime Biotech, Nanjing, China) as we previously described (Wang et al., 2018). Cells were stained with 20 μmol/L DCFH-DA in a dark room for 30 min and the fluorescence density of DCFH-DA was calculated by a fluorescence spectrometer (HTS 7000, Perkin Elmer, Boston, MA, United States).

The levels of ROS in the hippocampi of rat pups were measured using an ROS ELISA detection kit (Jianglai Biotech, Shanghai, China) according to the manufacturer’s instructions. The extracted supernatant of hippocampus tissue samples was added to the ELISA scale, reacted with conjugate for 1 h at 37°C, and then reacted with substrate for 20 min at 25°C followed by stop solution. Then, the fluorescence absorptions were measured by a microplate reader (HTS 7000, Perkin Elmer, Boston, MA, United States) at 450 nm immediately. The concentration of ROS was calculated corresponding to the mean absorbance from the standard curve.

### Hematoxylin and Eosin (HE) Staining

The 4-μm thick brain slices were deparaffinized and immersed in distilled water prior to hematoxylin staining. Then they were washed up in distilled water, dehydrated gradually using an alcohol gradient, then immersed in eosin for staining, dehydrated and vitrified again, and finally covered with resinene. The pyramidal neurons of the hippocampal CA1 region in each slice were observed with light microscope (Carl Zeiss, Oberkochen, Germany) by an observer blinded to the experiment. The criteria for neuronal death and injury were as follows: neuronal arrangement in sparce and disorder, cell shrinkage, morphologically pink cytoplasm, and pyknotic nuclei.

### Mitochondrial Membrane Potential Assay

The SH-SY5Y (5 × 10^5^ cells/well) and HT22 (5 × 10^5^ cells/well) cells were seeded in a 6-well culture plate for 24 h, then exposed to sevoflurane at concentrations of 4 and 8% for 12 h in the presence or absence of 3AB or NAC. As we previously described (Wang et al., 2018), the cells were collected and stained with JC-1 according to manufacturer’s instruction (Beyotime Biotech, Nanjing, China). Then, a group of cells were analyzed using flow cytometry (NoVoCyte, Hangzhou, China) while another group of cells were observed under a fluorescence microscope (Olympus IX71, Tokyo, Japan).

### Transfection of Small Interfering RNA (SiRNA)

For RNA interference experiments, SH-SY5Y cells (5 × 10^5^ cells/ml) and HT22 cells (5 × 10^5^ cells/ml) were seeded in a 6-well culture plate for 24 h. According to the manufacturer’s instructions, small interfering (SiRNA) was transfected to knock down PARP-1 by using Lipofectamine 3000 (Invitrogen, United States). The SiRNA targeting PARP-1 in SH-SY5Y cells and HT22 cells was 5′-GCAAAGGCCAGGAUGGAAUTT-3′ and 5′-CCAUGUUCGAUGGGAAAGUTT-3′, respectively. After 48 h of interference, cells were treated with sevoflurane at concentrations of 4 and 8% for 12 h, and the efficacy of target gene knockdown was evaluated by western blotting.

### Western Blotting Analysis

The SH-SY5Y (5 × 10^5^ cells/well) and HT22 (5 × 10^5^cells/well) cells were seeded in a 6-well cell culture plate for 24 h, and the primary hippocampal neurons (7 × 10^5^ cells/well) were seeded in a 6-well cell culture plate for 7 days. Cells were treated with sevoflurane at indicated times and concentrations in the presence or absence of 3AB, NAC, or PAPR-1 SiRNA. Nuclear and cytoplasmic proteins were isolated using a Nuclear and Cytoplasmic Protein Extraction Kit. Samples from cells and hippocampi were homogenized in an ice-cold RIPA buffer. Each homogenate was centrifuged at 1000 *g* for 10 min at 4°C to obtain the pellets containing nucleus and the supernatants including cytoplasm. After collecting the supernatant, the remaining cell pellets were added to nuclear protein extraction reagent to collect the supernatant containing nuclear proteins. The protein concentrations were quantified using a BCA Protein Assay Kit. Western blotting analysis was performed as described in our previous studies (Feng et al., 2016). Equal amounts of protein (20–30 μg) from cell lysates were separated by electrophoresis on 6–12% sodium dodecyl sulfate-polyacrylamide gel electrophoresis (SDS-PAGE) gels and transferred onto polyvinylidene difluoride (PVDF) membranes (Millipore, Billerica, MA, United States). The PVDF membranes were incubated at 4°C overnight with the following antibodies: anti-PARP-1 (1:1000), anti-PAR polymer (1:1000), anti-AIF (1:1000), anti-8-OHdG (1:1000), anti-γH2AX (1:1000), anti-ATM (1:1000), anti-p-ATM (1:1000), anti-β-actin (1:1000), and anti-Histone-3 (1:1000). Then, the membranes were incubated with horseradish-peroxidase-conjugated goat anti-rabbit IgG (1:1000) or horseradish-peroxidase-conjugated anti-mouse (1:1000) for 2 h at room temperature. Western blots were repeated for five independent experiments. The blots were visualized using an enhanced chemiluminescence kit and the blot densities measured using Image J software. Quantification of the blots was determined as the ratio of proteins to that of the loading control β-actin or histone.

### Immunofluorescence Staining

The SH-SY5Y (4 × 10^5^ cells/well) and HT22 (4 × 10^5^ cells/well) cells were seeded onto culture dishes with diameters of 3 cm and cultured for 24 h, then the cells were treated with sevoflurane at a concentration of 8% for 12 h in the presence or absence of 3AB or NAC. As we previously described (Wang et al., 2018), cells were fixed in 4% paraformaldehyde and permeabilized with 1% TritonX-100 for 10 min. After being blocked with 1% bovine serum albumin (BSA), the cells were incubated with anti-AIF antibody (1:500), followed by a secondary antibody for 1 h, then incubated with Hoechst 33342 following the manufacturer’s instructions. Finally, the cells were read using a confocal microscope (FV1000, Olympus, Japan).

### Morris Water Maze Tests

Morris water maze (MWM) was used to test spatial memory performance from postnatal day 35 to postnatal day 40 in rats that had been previously exposed with or without sevoflurane at postnatal day 7. The protocols of MWM were performed as we previously described (Feng et al., 2016). In brief, rats were placed in a large circular black paint tank with an inner diameter of 180 cm and filled with opaque water kept at a temperature of 25 ± 1°C. A platform was placed at 1.5 cm below the water surface by creating a nearly invisible platform to the background. Geometric objects with contrasting colors were set at remote ends of the water as references. The rat was gently released into the water with its nose against the wall from one of the four preplanned starting positions (North, South, East, or West). Spatial memory was assessed by recording the latency time for the rats to escape from the water onto a submerged escape platform during the learning phase. Learning trials were conducted over five consecutive days with four trials per day. On each of these trials, rats were allowed to find the hidden platform within 120 s, and the trainer, who was blinded to the group conditions, would guide them to the platform when the rats failed to find the platform, then the rats were left on the platform for 20 s for learning. Escape latency that represented the average time for rats to find the platform of four trials in a single day was recorded. In the probe trial, 24 h after the learning phase, the rats swam freely in the water tank without the platform for a single 120 s, and the time spent in the quadrant of the original platform was recorded. Monitoring was performed with a video tracking system (Noldus Ltd., EthoVision XT, Holland).

### Statistical Analysis

SPSS software version 24.0 (SPSS, Inc. IBM Corporation) was used for all statistical analyses. The normality of values was tested with Shapiro–Wilk normality test. Statistical differences between two groups of data were analyzed with a two-tailed unpaired *t*-test. One-way ANOVA and two-way ANOVA were used to investigate the differences in more than two groups, followed by an LSD test when appropriate. Behavioral studies were analyzed using two-way analysis of variance (ANOVA) with repeated measures, followed by the Bonferroni multiple comparison test. Experimental data were expressed as mean ± SD. A *P*-value of less than 0.05 was considered as statistically significant.

## Results

### Sevoflurane Inhibited Cell Viabilities and Induced Neuronal Cell Death

**FIGURE 1:**
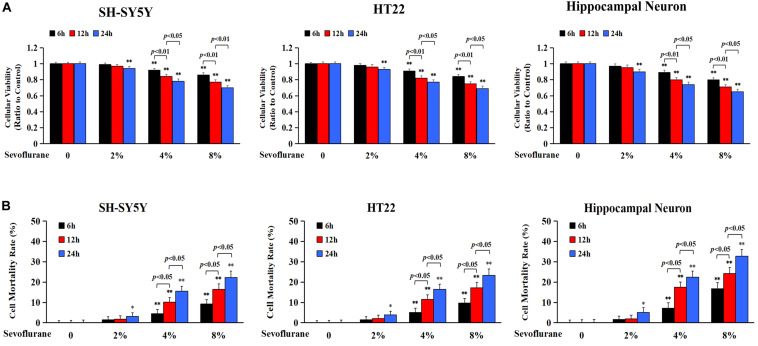
Sevoflurane inhibited cell viabilities and induced neuronal cell death. Human SH-SY5Y neuroblastoma cells, mouse hippocampal HT22 cells, and neonatal rat primary hippocampal neurons were treated with sevoflurane at concentrations of 2, 4, and 8% for 6, 12, and 24 h, respectively. **(A)** MTT assay showed that after sevoflurane exposure, cellular viabilities decreased significantly in a concentration- and time-dependent manner. **(B)** LDH release assay demonstrated that sevoflurane exposure significantly increased the rate of cell death in a concentration and time-dependent manner. Compared with the control group, ^∗^*p* < 0.05, ^∗∗^*p* < 0.01; Significant differences were shown when sevoflurane was exposed for 6 h *vs.* 12 h (*p* < 0.01), and for 12 h *vs.* 24 h (*p* < 0.01). Data are represented as mean ± SD from five independent experiments.

Previous studies have reported a cytotoxic effect caused by sevoflurane (Andropoulos, 2018) and 4.1% sevoflurane exposure for 6 h was found to induce cell death in H4 human neuroglioma cells (Zhou et al., 2016). To evaluate the toxicity of sevoflurane on neuronal cells, human SH-SY5Y cells, mouse HT22 cells, and neonatal rat primary hippocampal neurons were treated with 2, 4, and 8% sevoflurane for 6, 12, and 24 h, respectively, and cellular viabilities were tested using MTT assay. Human SH-SY5Y cells are human neuroblastoma cells sharing neuron-like properties in morphology and neurochemistry that expressed markers indicative of immature neurons. The mouse hippocampal HT22 cells are most similar to undifferentiated neural stem cells, which fits perfectly to evaluate the toxicity caused by oxidative stress. As shown in Figure 1A, compared with the control group, cellular viabilities decreased significantly in SH-SY5Y cells, HT22 cells, and hippocampal neurons when exposed to 2% sevoflurane for 24 h, and 4 and 8% sevoflurane for 6 h. Moreover, cellular viabilities were further decreased when neuronal cells exposed to 4 and 8% sevoflurane were prolonged to 12 and 24 h. Then, we examined whether sevoflurane induced neuronal cell death by using LDH release assay on the basis that cell membrane integrity was compromised in dying cells that released a quantitated amount of LDH. Compared with the control group, the cell death ratio of SH-SY5Y cells, HT22 cells, and hippocampal neurons increased significantly when they were exposed to 2% sevoflurane for 24 h, and 4 and 8% sevoflurane for 6 h, which became more aggravated with extended exposure duration (Figure 1B). Therefore, these data suggested that sevoflurane exposure inhibited cellular viabilities and induced neonatal neuronal cell death, in a concentration- and time-dependent manner.

### Parthanatos Contributed to Sevoflurane-Induced Neuronal Cell Death

**FIGURE 2:**
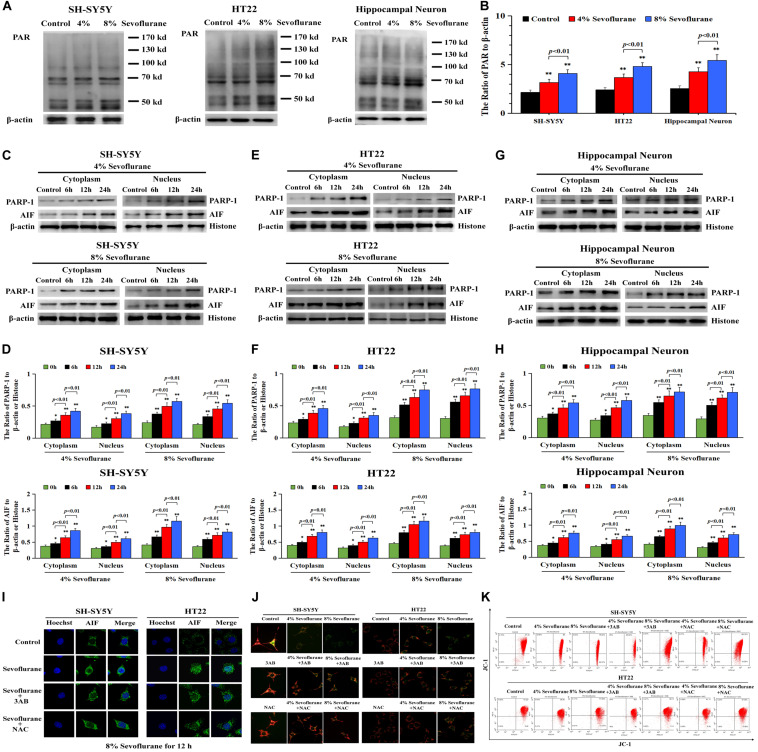
Sevoflurane induced changes of Parthanatos-related proteins and mitochondrial membrane potential in neuronal cells. **(A,B)** Western blotting and quantitative analysis of cytoplasmic PAR polymer after 4 and 8% sevoflurane exposure for 12 h in SH-SY5Y cells, HT22 cells, and hippocampal neurons. Compared with control group, ^∗∗^*p* < 0.01; compared with 4% sevoflurane, 8% sevoflurane significantly increased the ratio of PAR polymer to β-actin. **(C–H)** Western blotting and quantitative analysis of PAPR-1 and AIF both in cytoplasm and nucleus in SH-SY5Y cells, HT22 cells, and hippocampal neurons after 4 and 8% sevoflurane exposure for 6, 12, and 24 h. Compared with the control group, ^∗^*p* < 0.05, ^∗∗^*p* < 0.01. Significant differences in the ratio of PARP-1 and AIF to β-actin or histone were detected when sevoflurane was exposed for 6 h *vs.* 12 h (*p* < 0.01), and for 12 h *vs.* 24 h (*p* < 0.01). **(I)** Confocal microscopy observed that AIF accumulated in the nucleus of SH-SY5Y cells and HT22 cells after 8% sevoflurane exposure for 12 h, which were obviously alleviated by PARP-1 inhibitor 3AB at 500 μmol/L or antioxidant NAC at 5 mmol/L. **(J)** Fluorescence microscopy with JC-1 staining revealed that a concentration-dependent weakened red fluorescence and enhanced green fluorescence were markedly observed in SH-SY5Y cells and HT22 cells treated with 4 and 8% sevoflurane for 12 h, which were counteracted in the presence of PARP-1 inhibitor 3AB or antioxidant NAC. **(K)** Flow cytometry analysis showed that concentration-dependent decline of mitochondrial membrane potential after 4 and 8% sevoflurane for 12 h was attenuated in the presence of PARP-1 inhibitor 3AB or antioxidant NAC in SH-SY5Y cells and HT22 cells. Data are represented as mean ± SD from five independent experiments.

To clarify the mechanism accounting for the neuronal cell death caused by sevoflurane, we investigated whether Parthanatos was involved in sevoflurane-induced neuronal death. Given that one of the prominent features of Parthanatos is PARP-1 overactivation (Fatokun et al., 2014), we assayed the level of PAR polymer which is the product of PARP-1 activation. As shown by western blotting, when compared with the control group, the level of PAR polymer was increased significantly in SH-SY5Y cells, HT22 cells, and hippocampal neurons after being exposed to 4% sevoflurane for 12 h, which was further increased statistically when the concentration of sevoflurane was elevated to 8% (Figures 2A,B). Then, we examined sevoflurane-induced changes in the protein level of PARP-1. Western blotting showed that the expression of PARP-1 in neuronal cells was significantly upregulated in both the cytoplasm and nucleus following 4% sevoflurane exposure for 6 h. Moreover, the expression of PAPR-1 was further increased when cells were exposed to 8% sevoflurane for 6 h or sevoflurane with exposure time extended to 12 and 24 h (Figures 2C–H). These results indicated that sevoflurane induced PARP-1 hyperactivation and cytoplasmic PAR polymer accumulation in a concentration and time-dependent manner.

Consistent with western blotting showing that the level of AIF in the nuclei upregulated significantly (Figures 2C–H), the images from confocal microscopy further confirmed apparent AIF accumulation within the nuclei after sevoflurane exposure (Figure 2I). In normal conditions, AIF is located in the mitochondrial, which is the target of the PAR polymer during the process of Parthanatos (Fatokun et al., 2014), but when mitochondrial membrane potential was declined due to excess accumulation of PAR polymer, AIF is released from the mitochondria and translocated into the nucleus, leading to cell death (Fatokun et al., 2014). We thus indicated that sevoflurane-induced mitochondrial damage might be associated with cytoplasmic PAR polymer accumulation. Then, sevoflurane-induced damage in mitochondria was examined by using JC-1 staining. JC-1 is a probe that emits red fluorescence when accumulating in healthy mitochondria and green fluorescence in damaged mitochondria. As shown in Figure 2J, we found that the red fluorescence weakened and green fluorescence enhanced obviously when the neuronal cells were exposed to 4 and 8% sevoflurane. Meanwhile, flow cytometry analysis further proved the concentration-dependent decline of mitochondrial membrane potential after sevoflurane exposure in neuronal cells (Figure 2K), indicating that sevoflurane-induced mitochondrial depolarization was regulated by PAR polymer in response to PARP-1 activation. Therefore, these results suggested that sevoflurane exposure resulted in hyperactivation of PARP-1 and resultant accumulation of cytoplasmic PAR polymer, leading to mitochondrial dysfunction and AIF nuclear translocation.

**FIGURE 3:**
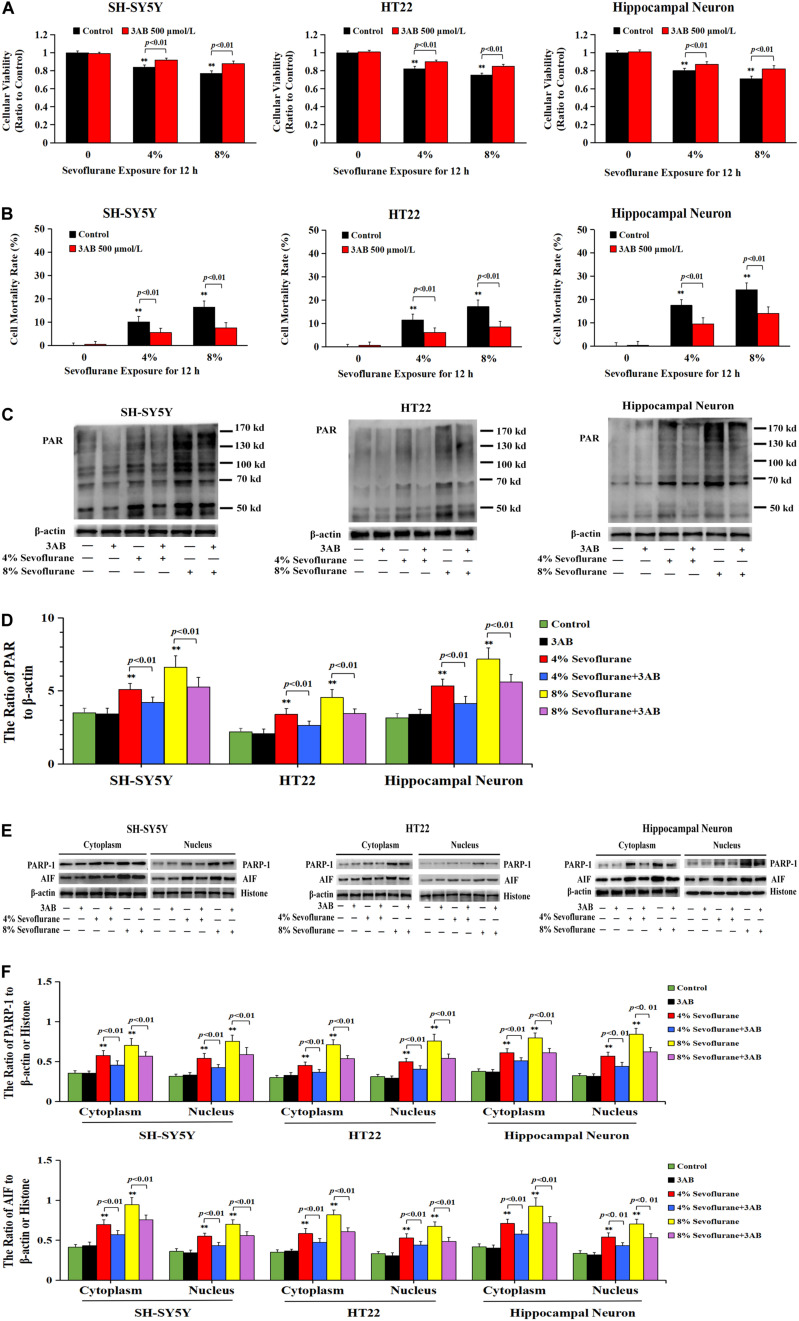
Pharmacological inhibition of PARP-1 with 3AB prevented sevoflurane-induced cytotoxicity in neuronal cells. **(A)** MTT analysis showed that PARP-1 inhibitor 3AB at 500 μmol/L significantly reversed the decline of cell viabilities induced by 4 and 8% sevoflurane for 12 h in SH-SY5Y cells, HT22 cells, and hippocampal neurons. **(B)** LDH release assay showed that 3AB markedly rescued cell death caused by 4 and 8% sevoflurane exposure for 12 h in SH-SY5Y cells, HT22 cells, and hippocampal neurons. **(C–F)** Western blotting and quantitative analysis showed that upregulation of cytoplasmic PAR polymer, PARP-1, and AIF both in cytoplasm and nucleus induced by 4 and 8% sevoflurane for 12 h were significantly inhibited when pretreated with 3AB in SH-SY5Y cells, HT22 cells, and hippocampal neurons. Compared with the control group, ^∗∗^*p* < 0.01; Compared with sevoflurane group, pretreatment of 3AB prior to sevoflurane significantly decreased the ratio of PAR, PAPR-1, and AIF to β-actin or histone (*p* < 0.01). Data are represented as mean ± SD from five independent experiments.

**FIGURE 4:**
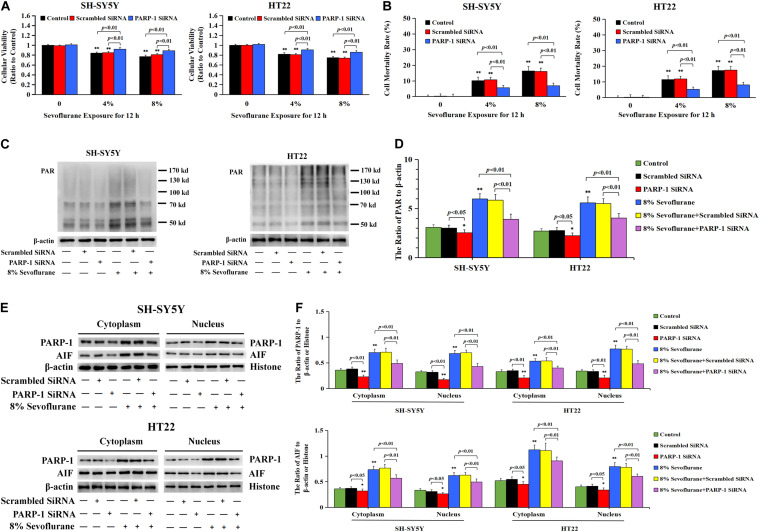
Knockdown the level of PARP-1 with SiRNA prevented sevoflurane-induced cytotoxicity in SH-SY5Y cells and HT22 cells. **(A)** MTT analysis demonstrated that knockdown of the level of PARP-1 with SiRNA prior to 4 and 8% sevoflurane for 12 h significantly prevented sevoflurane-induced reduction of cell viabilities in SH-SY5Y cells and HT22 cells. **(B)** LDH release assay demonstrated that knockdown of PARP-1 with SiRNA prior to 4 and 8% sevoflurane for 12 h markedly rescued sevoflurane-induced cell death in SH-SY5Y cells and HT22 cells. **(C–F)** Western blotting and quantitative analysis showed that upregulation of cytoplasmic PAR polymer, PARP-1, and AIF both in the cytoplasm and nucleus induced by 8% sevoflurane exposure for 12 h were significantly inhibited when SH-SY5Y cells and HT22 cells were transfected with PARP-1 SiRNA. Compared with the control group, ^∗^*p* < 0.05, ^∗∗^*p* < 0.01; Compared with scrambled SiRNA group, the ratio of PAR, PARP-1, and AIF to β-actin or Histone in the neuronal cells transfected with PARP-1 SiRNA were significantly decreased (*p* < 0.05, *p* < 0.01); Compared to sevoflurane with or without scrambled SiRNA, the ratio of PAR, PAPR-1, and AIF to β-actin or histone in the neuronal cells transfected with PARP-1 SiRNA prior to sevoflurane exposure were significantly decreased (*p* < 0.01). Data are represented as mean ± SD from five independent experiments.

To verify the role of PARP-1 in sevoflurane-induced neuronal cell death, the cells were pretreated with PARP-1 specific inhibitor 3AB for 1 h and then exposed to sevoflurane for 12 h. MTT assay proved that prior administration of 3AB at 500 μmol/L significantly increased the viabilities of neuronal cells exposed to sevoflurane (Figure 3A). LDH analysis demonstrated the released quantities of LDH due to sevoflurane exposure was decreased obviously by pretreatment of 3AB in neuronal cells, indicating that 3AB could inhibit sevoflurane-induced neuronal cell death (Figure 3B). Moreover, western blotting revealed that 3AB significantly prevented sevoflurane-induced upregulation of PARP-1, cytoplasmic PAR polymer accumulation, and AIF nuclear translocation (Figures 3C–F). Images from confocal microscopy further confirmed that apparent AIF accumulation within the nuclei after sevoflurane exposure was significantly decreased by administration of 3AB (Figure 2I). Meanwhile, sevoflurane-induced decline of mitochondrial membrane potential was reversed obviously with pretreatment of 3AB (Figures 2J,K). Additionally, small interfering RNA was introduced to knock down PARP-1 in SH-SY5Y cells and HT22 cells to further confirm the role of PARP-1 in sevoflurane-induced neuronal death. Compared to sevoflurane with or without scrambled SiRNA, MTT assay and LDH analysis proved that sevoflurane-induced reduction in cellular viabilities and increase in cell death were counteracted when PARP-1 was knocked down by SiRNA (Figures 4A,B). Western blotting proved that either in the absence of sevoflurane or being treated 12 h with sevoflurane, the neuronal cells transfected with PARP-1 SiRNA had a lower level of PAPR-1, cytoplasmic PAR polymer, and nuclear AIF when compared with that in the control group or scrambled SiRNA group (Figures 4C–F).

Taken together, these results suggested that sevoflurane-induced death in neuronal cells was PARP-1 dependent, which was consistent with the criteria for the determination of Parthanatos.

### Sevoflurane Induced DNA Damage in Neuronal Cells

**FIGURE 5:**
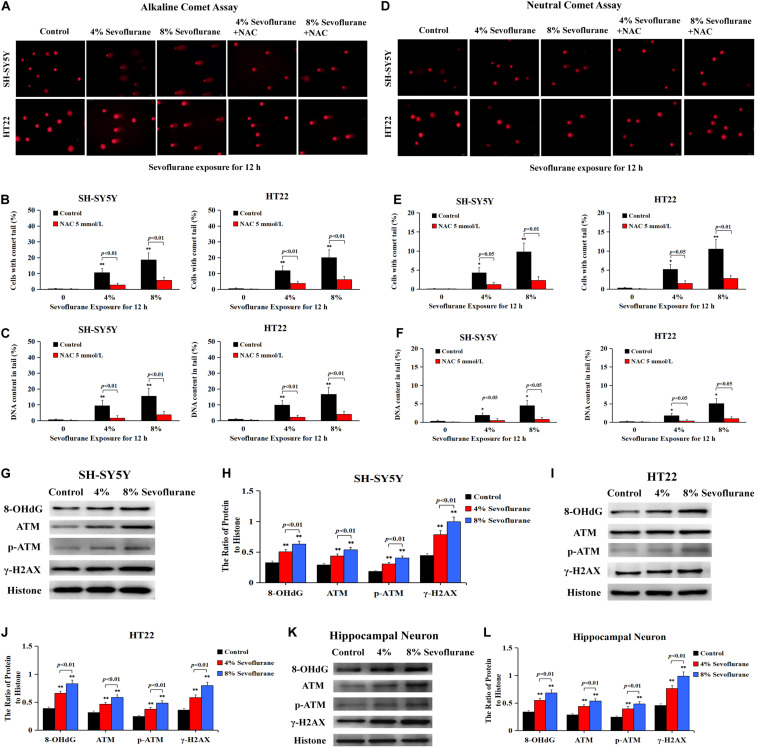
Sevoflurane induced DNA damage in neuronal cells. **(A)** Images of neuronal cells acquired from fluorescence microscopy after alkaline comet assay. SH-SY5Y cells and HT22 cells treated with 4 and 8% sevoflurane for 12 h showed more cells with comet tails than that in control group, and the higher concentration, the longer the comet tails. Pretreatment of neuronal cells with antioxidant NAC at 5 mmol/L markedly prevented sevoflurane-induced appearance of comet tails in SH-SY5Y cells and HT22 cells. **(B,C)** Statistical analysis of alkaline comet assay revealed that sevoflurane induced a significant increase in the percentage of cells with comet tails and DNA content within the tails (*p* < 0.01), which were markedly inhibited in the presence of NAC (*p* < 0.01). **(D)** Images of neuronal cells acquired from fluorescence microscopy after neutral comet assay. Compared with the control group, SH-SY5Y cells and HT22 cells treated with 4 and 8% sevoflurane for 12 h presented longer comet tails, in a concentration-dependent manner. However, the appearance of comet tails in SH-SY5Y cells and HT22 cells caused by sevoflurane were obviously prevented by pretreatment of NAC. **(E,F)** Statistical analysis of neutral comet assay demonstrated that sevoflurane induced a significant increase in the percentage of cells with comet tails and DNA content within the tails (*p* < 0.05, *p* < 0.01), which were markedly reversed by pretreatment of NAC (*p* < 0.05, *p* < 0.01). **(G–L)** Western blotting and quantitative analysis showed that 4 and 8% sevoflurane for 12 h concentration-dependently induced upregulation of DNA damage-related protein 8-OHdG, γH2AX, and p-ATM in SH-SY5Y cells, HT22 cells, and hippocampal neurons (*p* < 0.01). Compared with the control group, ^∗^*p* < 0.05, ^∗∗^*p* < 0.01. Data are represented as mean ± SD from five independent experiments.

Given that DNA damage is identified as the major initiator of Parthanatos (Wang et al., 2009; Fatokun et al., 2014; Lee et al., 2014) and PARP-1 is activated in response to different types of DNA damage, including single strand breaks (SSBs) and double strand breaks (DSBs) (Chaudhuri and Nussenzweig, 2017), we thus examined the changes of DNA damage after sevoflurane exposure in neuronal cells. Comet assay was performed to detect the DNA strand nicks and breaks. As shown in Figure 5A, alkaline comet assay, which detects DNA strand breaks of both SSBs and DSBs, observed that when neuronal cells were exposed to 4 and 8% sevoflurane for 12 h, the higher the concentrations, the more cells with comet tail. Statistical analysis revealed that sevoflurane exposure resulted in more cells with longer comet tails and DNA content within the tails (Figures 5B,C). Likewise, neutral comet assay, which detects only DSBs, showed that sevoflurane-treated neuronal cells have more DNA DSBs in a concentration-dependent manner (Figures 5D–F). Additionally, we observed the levels of proteins representing DNA damage markers, including 8-hydroxydeoxyguanosine (8-OHdG) which is a pivotal product of DNA base lesion caused by oxidative stress, and phosphorylation of histone variant H2AX at Ser139 (γH2AX) which is a sensitive and specific marker of DNA DSBs activated by phosphorylation of protein kinase ataxia telangiectasia mutated (ATM). Western blotting showed that SH-SY5Y cells, HT22 cells, and hippocampal neurons treated with sevoflurane exposure at concentrations of 4 and 8% for 12 h induced visible increases in the levels of 8-OHdG, γH2AX, and p-ATM (Figures 5G–L), indicating that sevoflurane exposure induced DNA damage, including both SSBs and DSBs, in a concentration-dependent manner. Therefore, on the basis that DNA damage is a causal factor leading to PARP-1 activation, we think that sevoflurane induced DNA damage and resultant PARP-1-dependent neuronal cell death, indicating that DNA damage is implicated in sevoflurane-induced neuronal cell Parthanatos.

### ROS Regulated Sevoflurane-Induced DNA Damage and Parthanatos in Neuronal Cells

**FIGURE 6:**
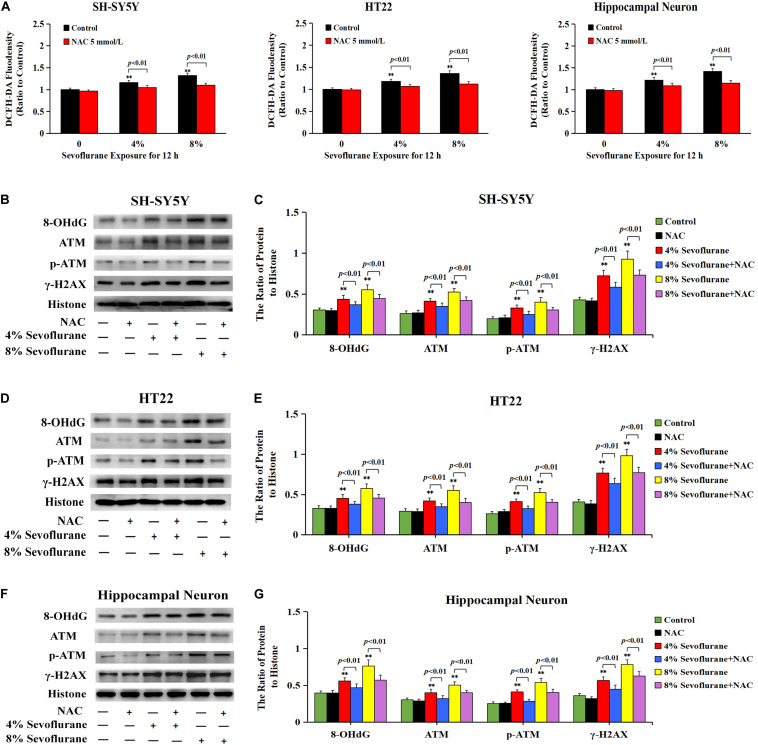
ROS contributed to sevoflurane-induced DNA damage in neuronal cells. **(A)** Fluorescence intensity using DCFH-DA showed that ROS was excessively generated in SH-SY5Y cells, HT22 cells, and hippocampal neurons when exposed to 4 and 8% sevoflurane for 12 h. Pretreatment of antioxidant NAC at 5 mmol/L significantly inhibited sevoflurane-induced overproduction of intracellular ROS. **(B–G)** Western blotting and quantitative analysis showed that 4 and 8% sevoflurane for 12 h significantly upregulated the levels of protein 8-OHdG, γH2AX, and p-ATM in SH-SY5Y cells, HT22 cells, and hippocampal neurons, which were markedly attenuated by pretreatment of NAC. Compared with the control group, ^∗∗^*p* < 0.01; Compared with sevoflurane group, pretreatment of NAC prior to sevoflurane exposure significantly decreased the ratio of 8-OHdG, γH2AX, and p-ATM to histone (*p* < 0.01). Data are represented as mean ± SD from five independent experiments.

To explore why sevoflurane exposure could induce DNA damage, we investigated the role of sevoflurane in regulating intracellular ROS levels because ROS is an intrinsic factor leading to DNA damage. The level of intracellular ROS was examined by using DCFH-DA following sevoflurane exposure. Compared with the control group, quantitative analysis of fluorescence intensity revealed that the levels of intracellular ROS were significantly increased with 4% sevoflurane and reached a higher level with 8% sevoflurane, however, pretreatment with antioxidant NAC at 5 mmol/L obviously counteracted sevoflurane-induced elevation of ROS in neuronal cells (Figure 6A), indicating that sevoflurane induced the overproduction of intracellular ROS in neuronal cells. Consistent with both alkaline and neutral comet assay that antioxidant NAC markedly attenuated sevoflurane-induced increase in the cells with comet tails (Figures 5A–F), western blotting showed that upregulation of 8-OHdG, γH2AX, and p-ATM caused by sevoflurane were obviously mitigated in the presence of NAC (Figures 6B–G). Collectively, these results suggested that ROS contributed to sevoflurane-induced DNA damage in neuronal cells.

**FIGURE 7:**
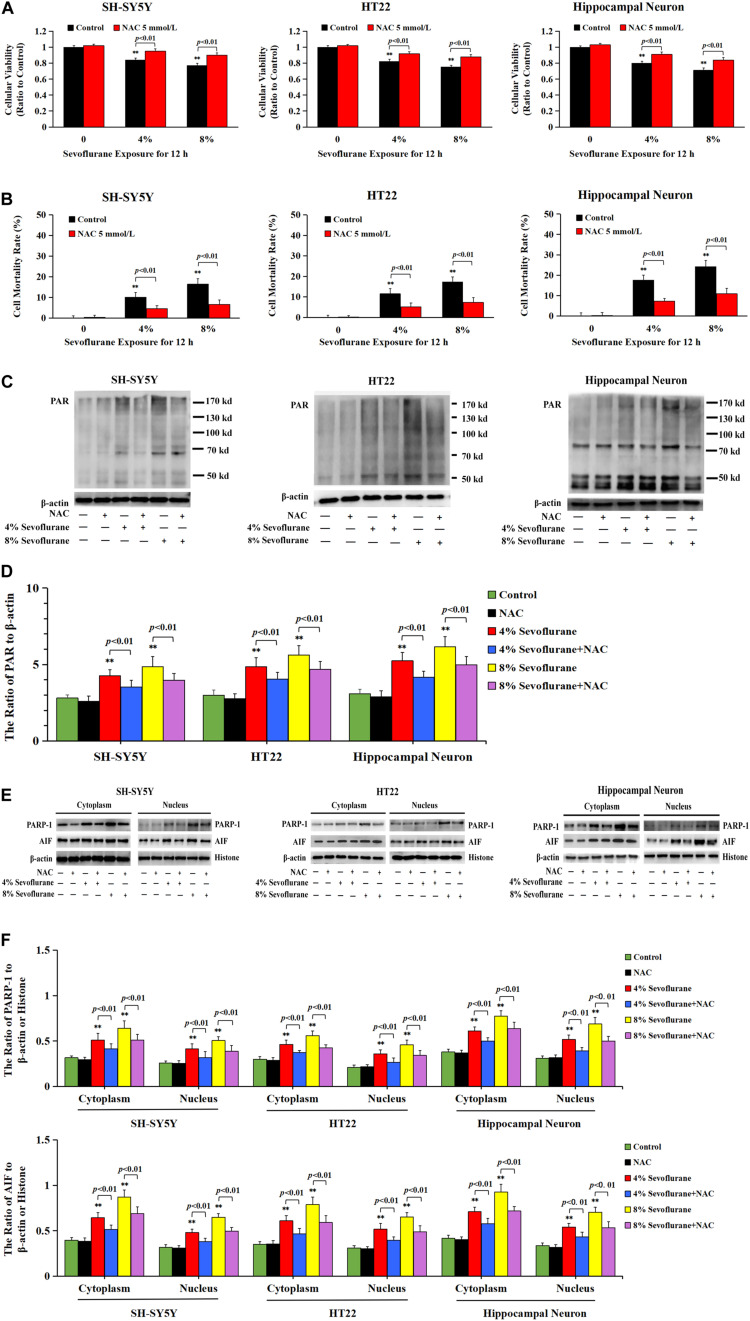
ROS regulated sevoflurane-induced Parthanatos in neuronal cells. **(A)** MTT analysis showed that reduction of cell viabilities induced by 4 and 8% sevoflurane for 12 h in SH-SY5Y cells, HT22 cells, and hippocampal neurons were markedly reversed by pretreatment of NAC. **(B)** LDH release assay showed that pretreatment of NAC markedly rescued cell death caused by 4 and 8% sevoflurane exposure for 12 h in SH-SY5Y cells, HT22 cells, and hippocampal neurons. **(C–F)** Western blotting and quantitative analysis showed that 4 and 8% sevoflurane for 12 h significantly upregulated the levels of cytoplasmic PAR polymer, PARP-1, and AIF both in cytoplasm and nucleus in SH-SY5Y cells, HT22 cells, and hippocampal neurons, which were markedly alleviated by pretreatment of NAC. Compared with control group, ^∗∗^*p* < 0.01; Compared with sevoflurane group, significant differences were shown in neuronal cells treated with NAC prior to sevoflurane exposure (*p* < 0.01). Data are represented as mean ± SD from five independent experiments.

Considering that the increase of intracellular ROS was found to be implicated in the occurrence of Parthanatos (Zheng et al., 2017; Wang et al., 2018), we then examined the role of ROS in sevoflurane-induced neuronal Parthanatos. MTT assay and LDH release analysis proved that sevoflurane-induced reduction of cellular viabilities and increase of neuronal cell death were significantly attenuated in the presence of NAC (Figures 7A,B). Furthermore, western blotting showed that sevoflurane-induced upregulation of PARP-1, PAR polymer, and nuclear AIF were obviously decreased with pretreatment of NAC (Figures 7C–F), which was similar to the inhibitory effect of 3AB (Figures 3C–F). Additionally, JC-1 staining and flow cytometry analysis proved that inhibition of ROS with NAC significantly prevented the decline of mitochondrial membrane potential caused by sevoflurane in neuronal cells, which accordingly suppressed sevoflurane-induced AIF accumulation within the nuclei, as shown by images from confocal microscopy (Figures 2I–K). Thus, these data indicated that ROS contributed to sevoflurane-induced neuronal cell Parthanatos.

### DNA Damage Accounted for ROS-Induced Neuronal Cell Parthanatos

**FIGURE 8:**
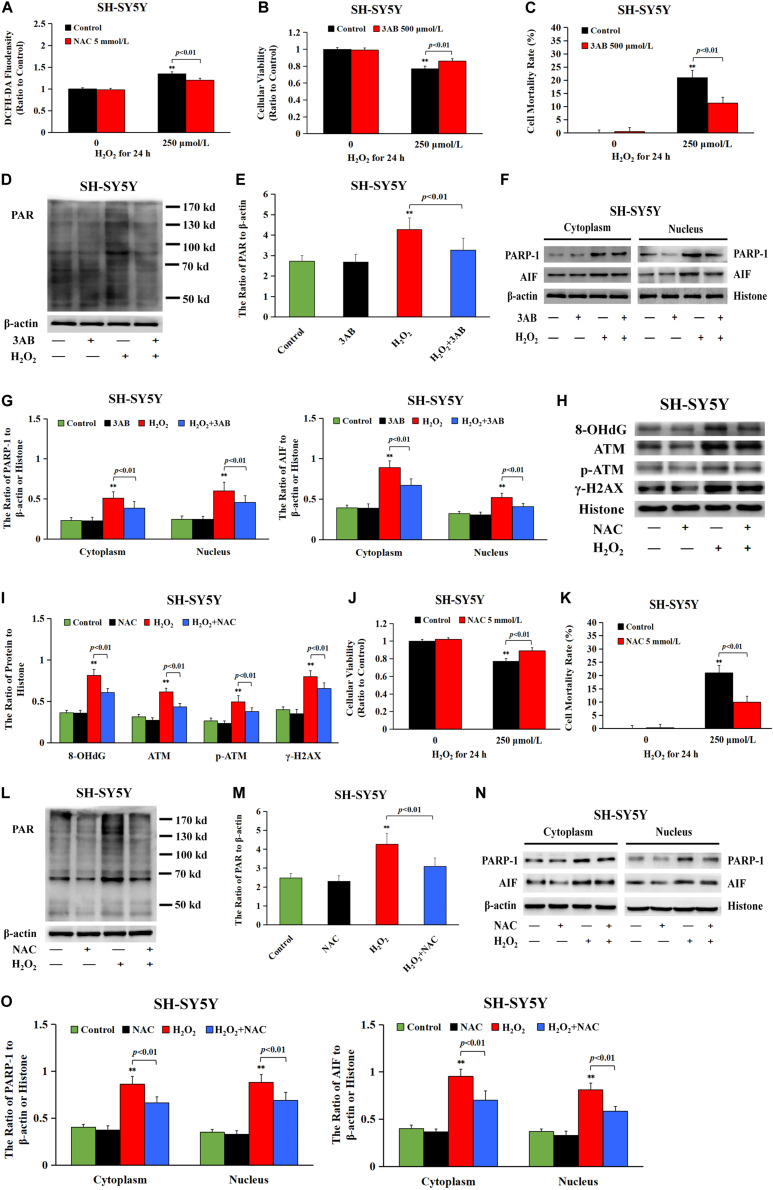
DNA damage participated in H_2_O_2_-induced neuronal cell Parthanatos. **(A)** Fluorescence intensity using DCFH-DA showed that H_2_O_2_ exposure at 250 μmol/L for 24 h significantly induced ROS production in SH-SY5Y cells, which was significantly inhibited by pretreatment with antioxidant NAC. **(B)** MTT analysis showed that H_2_O_2_-induced reduction in cell viability of SH-SY5Y cells was markedly reversed with pretreatment of PARP-1 inhibitor 3AB. **(C)** LDH release assay showed that H_2_O_2_-induced cell death in SH-SY5Y cells was obviously rescued with pretreatment of PARP-1 inhibitor 3AB. **(D–G)** Western blotting and quantitative analysis showed that H_2_O_2_-induced upregulation of cytoplasmic PAR polymer, PARP-1, and AIF both in cytoplasm and nucleus were obviously attenuated by pretreatment of 3AB in SH-SY5Y cells. **(H,I)** Western blotting and quantitative analysis showed that H_2_O_2_-induced upregulation of protein 8-OHdG, γH2AX, and p-ATM were obviously attenuated by pretreatment of NAC in SH-SY5Y cells. **(J)** MTT analysis showed that H_2_O_2_-induced reduction in cell viability of SH-SY5Y cells was markedly reversed in the presence of NAC. **(K)** LDH release assay showed that H_2_O_2_-induced cell death in SH-SY5Y cells was significantly rescued in the presence of NAC. **(L–O)** Western blotting and quantitative analysis showed that H_2_O_2_-induced upregulation of cytoplasmic PAR polymer, PARP-1, and AIF both in cytoplasm and nucleus were obviously attenuated by pretreatment of NAC in SH-SY5Y cells. Compared with the control group, ^∗∗^*p* < 0.01; Compared with H_2_O_2_ group, significant differences were shown in H_2_O_2_ group pretreated with 3AB or NAC (*p* < 0.01). Data were presented as mean ± SD from five independent experiments.

Given that intracellular ROS accounted for DNA damage and neuronal cell Parthanatos caused by sevoflurane exposure, hydrogen peroxide (H_2_O_2_) was used to verify the role of ROS-initiated DNA damage in Parthanatos of neuronal cells. DCFH-DA fluorescence intensity proved that H_2_O_2_ at a concentration of 250 μmol/L significantly triggered the overproduction of intracellular ROS in SH-SY5Y cells, which was inhibited by pretreatment of antioxidant NAC (Figure 8A). We found that H_2_O_2_-induced SH-SY5Y cell death and upregulation of PAR polymer accumulation, PARP-1, and nuclear AIF were all mitigated by pretreatment of 3AB, indicating that ROS induced SH-SY5Y cell Parthanatos (Figures 8B–G). Moreover, an overt increase in the levels of 8-OHdG, γH2AX, and p-ATM after H_2_O_2_ treatment were detected (Figures 8H,I), indicating that ROS induced DNA damage of both DNA SSBs and DSBs. On the contrary, pretreatment of antioxidant NAC not only prevented H_2_O_2_-induced DNA damage (Figures 8H,I), but also alleviated H_2_O_2_-induced cell death as well as upregulation of PARP-1, PAR polymer, and nuclear AIF (Figures 8J–O). Therefore, these results suggested that ROS-initiated DNA damage contributed to Parthanatos of neuronal cells.

### Sevoflurane Induced Neuronal Parthanatos in Hippocampus of Neonatal Rats

The brain growth of rat increases significantly during the first 2 weeks after birth (Satomoto et al., 2009). Previous studies have reported that single exposure of neonatal rats to 2–3% sevoflurane for 4–6 h induced widespread neuronal cell death and long-term cognitive deficits (Zhou et al., 2012; Liu et al., 2015; Perez-Zoghbi et al., 2017; Tang et al., 2018). Thus, in the present study, neonatal rat pups (P7) with 2.5% sevoflurane for 6 h were used to evaluate the developmental neurotoxicity of volatile anesthetics *in vivo*. Considering that the anesthesia procedure would induce hypoxia, hypercapnia, and hypoglycemia, all factors that may affect neuronal function, arterial blood gas analysis at the end of sevoflurane exposure was performed, showing no significant disturbances in oxygenation and blood glucose, but slightly elevated PaCO_2_ (lower than 60 mmHg) (Table 1). Given that PaCO_2_ among 60–100 mmHg neither increases neuronal cell death nor causes neuronal dysfunction in rats (Zhou et al., 2010; Yufune et al., 2016; Perez-Zoghbi et al., 2017), we are able to exclude the impact of hypercapnia on neuronal function in our findings.

**TABLE 1:** Arterial blood gas after 6 h of 2.5% sevoflurane or no anesthesia in P7 rat pups (*n* = 6 per group).

	Control	3AB	NAC	Sevoflurane	Sevoflurane+ 3AB	Sevoflurane+ NAC
pH	7.370.07	7.390.08	7.360.05	7.310.06	7.320.07	7.300.05
PaCO_2_ (mmHg)	44.74.3	42.33.9	47.05.5	57.36.6	54.25.6	59.57.8
PaO_2_ (mmHg)	273.219.9	269.016.9	277.021.1	252.316.1	256.517.0	248.014.4
BE (mmol⋅l^–1^)	0.30.2	−0.40.3	0.50.4	−2.51.0	−1.80.7	−2.81.1
Glucose (mmol⋅l^–1^)	5.20.8	5.51.0	5.00.9	4.10.7	4.40.8	4.20.6

*Data are presented as mean ± SD. PaCO_2_, partial pressure of arterial carbon dioxide; PaO_2_, partial pressure of arterial oxygen; BE, base excess.*

**FIGURE 9:**
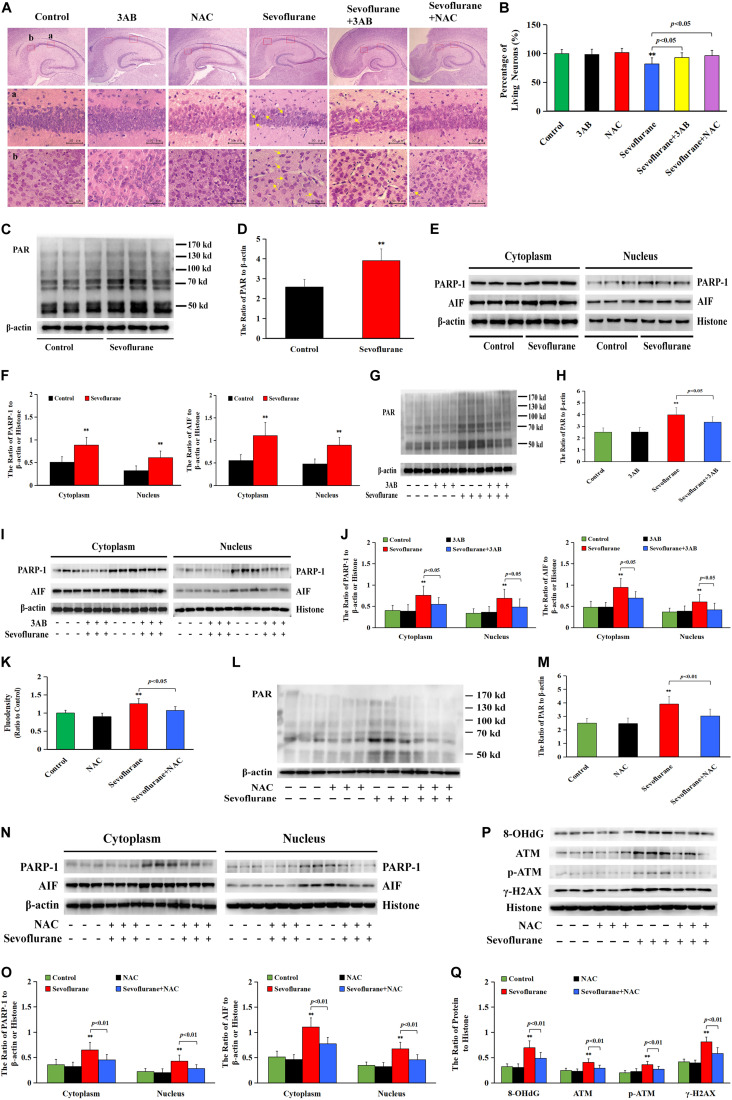
Sevoflurane induced neuronal Parthanatos in hippocampus of neonatal rats **(A)** Representative images of hippocampal neurons stained by hematoxylin and eosin (HE) staining in P7 rat pups at 7 days after 2.5% sevoflurane exposure for 6 h. Scale bar = 50 μm. Compared to the control group, sevoflurane induced pyramidal neuron death (yellow arrow) in the hippocampal CA1 region, presenting in sparce and disordered arrangement of neurons, morphologically cell shrinkage, pink cytoplasm, and pyknotic nuclei, which were rescued by pretreatment with PAPR-1 inhibitor 3AB at 30 mg/kg or antioxidant NAC at 90 mg/kg intraperitoneally. **(B)** Statistical analysis of living neurons in hippocampal CA1 regions showed that pretreatment of 3AB or NAC significantly rescued sevoflurane-induced reduction of living neurons. **(C–J,L–O)** Western blotting and quantitative analysis showed that 2.5% sevoflurane for 6 h significantly upregulated the levels of cytoplasmic PAR polymer, PAPR-1, and AIF both in the cytoplasm and nucleus in the hippocampi of rat pups, which were markedly attenuated by pretreatment of 3AB or NAC. **(K)** The levels of ROS using ELISA method showed that 2.5% sevoflurane for 6 h significantly increased ROS overproduction in hippocampus of rat pups, which were markedly prevented by pretreatment with antioxidant NAC. **(P,Q)** Western blotting and quantitative analysis showed that 2.5% sevoflurane for 6 h significantly upregulated the levels of 8-OHdG, γH2AX, and p-ATM in hippocampi of rat pups, which were markedly attenuated by pretreatment of NAC. Compared with the control group, ^∗∗^*p* < 0.01; Compared with sevoflurane group, significant differences were shown in rat pups pretreated with 3AB or NAC prior to sevoflurane exposure (*p* < 0.05, *p* < 0.01). Data are represented as mean ± SD from five independent experiments.

As is shown in Figure 9A, HE staining found neuronal death or injury that appeared on day 7 after sevoflurane anesthesia, featured by neuronal arrangement in sparce and disorder, cell shrinkage, morphologically pink cytoplasm, and pyknotic nuclei, presenting with only 82% of the pyramidal neurons alive in hippocampal CA1 region (Figure 9B), indicating that 2.5% sevoflurane exposure for 6 h induced significant hippocampal neuronal death in neonatal rats. Meanwhile, western blotting analysis showed that sevoflurane exposure significantly upregulated the levels of PARP-1, PAR polymers, and nuclear AIF in the hippocampus of rat pups at 6 h after anesthesia (Figures 9C–F). In contrast, pretreatment with PAPR-1 inhibitor 3AB not only significantly inhibited neonatal morphological changes caused by sevoflurane exposure and markedly increased the number of surviving pyramidal neurons in hippocampal CA1 region (Figures 9A,B), but also reversed sevoflurane-induced upregulation of PARP-1, PAR polymers, and nuclear AIF, indicating that 2.5% sevoflurane exposure for 6 h induced hippocampal neuronal Parthanatos in the developing brain (Figures 9G–J). Further, we found that sevoflurane exposure induced a significant increase of ROS in the hippocampus of pups at 6 h after anesthesia (Figure 9K). Pretreatment with NAC significantly inhibited sevoflurane-induced overproduction of ROS, upregulation of Parthanatos-related proteins, and increase in the levels of 8-OHdG, γH2AX and p-ATM, as well as neuronal death in the hippocampus (Figures 9A,B,K–Q). Therefore, these results suggested that sevoflurane induced hippocampal neuronal Parthanatos triggered by DNA damage in neonatal rats via an increase of ROS.

### Spatial Memory Deficits Induced by Early Exposure to Sevoflurane in Rats Are Alleviated by Pretreatment of 3AB or NAC

**FIGURE 10:**
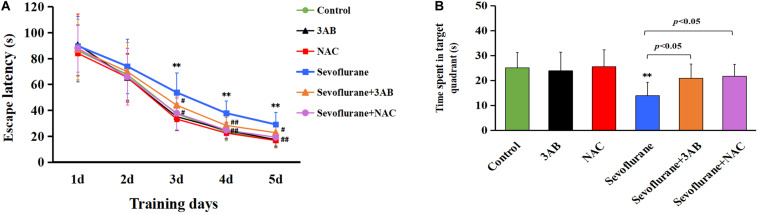
The spatial memory deficits induced by early exposure to sevoflurane in rats are alleviated by pretreatment of 3AB or NAC. Morris water maze (MWM) was used to test spatial memory performance from postnatal day 35 to postnatal day 40. **(A)** Escape latency was significantly longer in the sevoflurane group at training day 3, day 4, and day 5 than that in the control group, whereas the rats exposed to sevoflurane in the presence of 3AB or NAC showed markedly shorter latencies when compared with the sevoflurane group. **(B)** Statistical analysis showed that the rats exposed to sevoflurane spent significantly less time in the target quadrant during the probe trail on day 6 than in the control group, whereas the rats exposed to sevoflurane in the presence of 3AB or NAC spent significantly more time crossing the target quadrant. Compared with the control group, ^∗∗^*p* < 0.01; Compared with sevoflurane group, significant differences were shown in sevoflurane group pretreated with 3AB or NAC, ^#^*p* < 0.05, ^##^*p* < 0.01. Data are represented as mean ± SD (*n* = 6 rats per group).

Given that the hippocampus is associated with spatial learning and memory, spatial memory performance was determined using the MWM test on postnatal day 35 in rats that had previously received sevoflurane exposure on postnatal day 7 (Figure 10A). All rats had a significantly reduced latency to find the hidden platform as training progressed, indicating that the rats were learning from day-by-day practice. However, we found that exposure of 2.5% sevoflurane to P7 rats for 6 h in different groups caused significantly different performances in the behavioral test. The rats exposed to sevoflurane significantly prolonged the latency to locate the hidden platform on training day 3, 4, and 5, and less time spent in the target quadrant during the probe trial on day 6 than the control group (Figures 10A,B). Compared with the sevoflurane group, the rats treated with 3AB or NAC prior to sevoflurane exposure showed significantly shorter latencies to locate the hidden platform and significantly prolonged the swimming time in the target quadrant during the probe trial (Figures 10A,B). These results indicated that inhibition of PARP-1 or ROS could attenuate spatial memory impairment induced by early exposure to sevoflurane.

## Discussion

**FIGURE 11:**
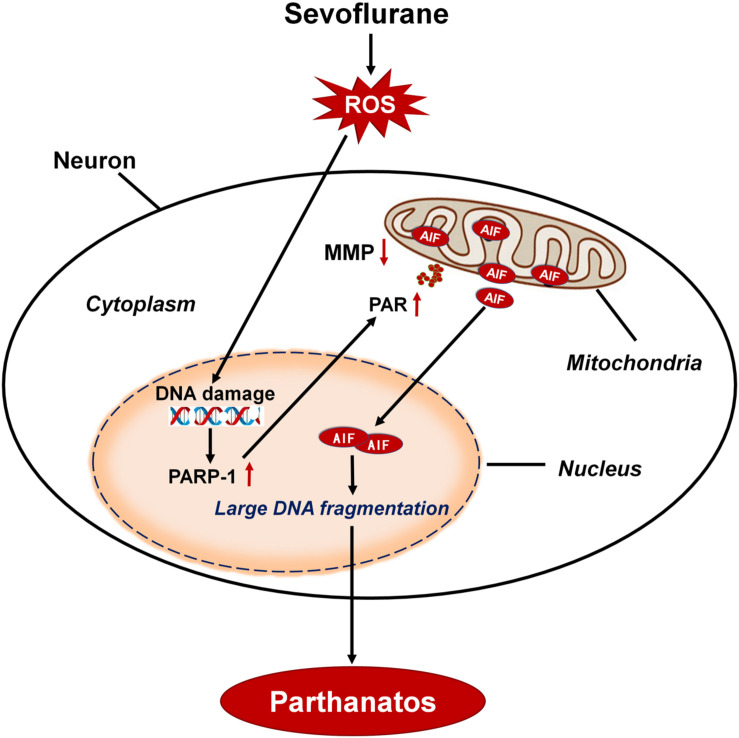
Schematic demonstrating the role of oxidative DNA damage in sevoflurane-induced neuronal cell Parthanatos. In the developing brain, sevoflurane exposure results in overproduction of intracellular reactive oxygen species (ROS). ROS-induced oxidative stress contributes to DNA damage, which is associated with neuronal cell death. Massive DNA damage initiates excessive PARP-1 activation and subsequent cytoplasmic PAP polymer accumulation which results in mitochondrial depolarization, leading to the AIF translocation from mitochondria to nucleus. Therefore, sevoflurane contributes to overproduction of ROS and resultant DNA damage, leading to PARP-1-dependent cell death (Parthanatos).

Minimal alveolar concentrations (MAC) of sevoflurane in neonates is 3.3% and in infants of 1–6 months of age is 3.2% (Lerman et al., 1994). The common concentrations of sevoflurane used in pediatric patients are about 0.5–2 MAC. So, we chose 2.5% sevoflurane (0.75 MAC) in our neonatal rat study and 4 or 8% sevoflurane (1.2 or 2.4 MAC) *in vitro* study, which are clinically relevant concentrations of sevoflurane. In this study, we found that sevoflurane exposure triggered neuronal cell death in SH-SY5Y cells, HT22 cells, primary hippocampal neurons, and the hippocampus of neonatal rats. This cell death occurs as a result of cytoplasmic PAR polymer accumulation and AIF nuclear translocation secondary to PAPR-1 hyperactivation, consistent with the criteria for Parthanatos. Of note, neuronal DNA damage induced by sevoflurane exposure was associated with the overproduction of intracellular ROS. Conversely, inhibition of intracellular ROS alleviated DNA damage, which suppressed sevoflurane-induced neonatal neuronal Parthanatos. Therefore, our results demonstrated that, after sevoflurane exposure, DNA damage caused by the overproduction of intracellular ROS played a crucial role in PAPR-1-dependent cell death, linking Parthanatos to sevoflurane-induced neuronal cell death in the developing brain (Figure 11).

Preclinical and clinical studies have shown the neurotoxic effects of sevoflurane on the developing brain, which may cause widespread neurodegeneration and long-term neurocognitive defects (Fang et al., 2012; Feng et al., 2012; Olsen and Brambrink, 2013; Lin et al., 2014; Makaryus et al., 2015; Diaz et al., 2016). Neuronal cell death has been considered a significant process of developmental neurotoxicity following sevoflurane exposure. Although sevoflurane was found to induce multiple forms of cell death, including apoptosis and autophagy (Zheng et al., 2013; Wang et al., 2019), the present study showed that Parthanatos accounted for the neonatal neuronal cell death caused by sevoflurane exposure. PAR polymer, generated as a product of PARP-1 hyperactivation, is thought to be an executioner of Parthanatos (Andrabi et al., 2006; Fatokun et al., 2014). We found that sevoflurane-induced neuronal cell death decreased significantly in neuronal cells and hippocampi of neonatal rats when excess PAR polymer was alleviated by PARP-1 inhibitor 3AB or PARP-1 knockdown with SiRNA, which was in accordance with the determination of Parthanatos (Zhao et al., 2015). Recognized as a death effector in Parthanatos, AIF is originally located in the mitochondria, which links PAR polymer toxicity to Parthanatos. It has been proven that cytoplasmic PAR polymer acted as an upstream regulator of mitochondria in the process of Parthanatos, which signaled to induce mitochondrial depolarization and AIF nuclear translocation, leading to cell death (Wang et al., 2009; Fatokun et al., 2014). Consistently, the present study showed that inhibition of cytoplasmic PAR polymer accumulation with 3AB or PARP-1 SiRNA not only markedly suppressed sevoflurane-induced mitochondrial depolarization, but also mitigated translocation of AIF into the nucleus both *in vitro* and *in vivo*. Therefore, these data indicated that sevoflurane exposure induced neuronal cell Parthanatos in the developing brain, which was mediated by PAR polymer accumulation through hyperactivation of PARP-1 and set in motion the process of cell death triggered by AIF nuclear translocation.

PARP-1, a multifunctional nuclear enzyme focused on the maintenance of genomic stability, is responsible for different types of DNA damage, including DNA base excision repair (BER), DNA single strand breaks (SSBs), and double strand breaks (DSBs) (Smith, 2001; Hong et al., 2004; Krietsch et al., 2013; Ray Chaudhuri and Nussenzweig, 2017). DNA damage is found to be associated with the pathogenesis of various diseases, such as neurological disorders, ischemia reperfusion injury, and cancer (Mocanu and Yellon, 2003; Rass et al., 2007; Stratton et al., 2009). It has been demonstrated that knockdown of DNA damage repair related genes contributed to abnormal neurodevelopment and neuronal cell death in mouse embryos (Kisby et al., 2013), indicating that DNA damage repair played a vital role in the early phase of neurodevelopment. In the present study, sevoflurane-induced DNA strand nicks and breaks were obviously detected by alkaline and neutral comet assay, suggesting that sevoflurane exposure resulted in both DNA SSBs and DNA DSBs. DNA DSBs triggered γH2AX by activating ATM at the site of DNA damage (Bonner et al., 2008). Consistent with the results from comet assay, we observed in neuronal cells and hippocampi of neonatal rats that sevoflurane exposure was associated with increased levels of γH2AX and p-ATM. In support, neuronal death following stroke and traumatic brain injury is found to stem in part from the overactivation of PARP-1 due to massive DNA damage (Andrabi et al., 2008; Ross and Truant, 2017). Hoch et al. proved that DNA damage caused by defective DNA SSBs repair in human and mice cerebella ataxia induced hyperactivity of PARP-1, and subsequently resulted in neuronal cell Parthanatos (Hoch et al., 2017). Collectively, these data suggested that sevoflurane induced DNA damage and resultant hyperactivation of PARP-1, leading to neonatal neuronal cell Parthanatos.

Intracellular ROS-induced oxidative stress played a crucial role in DNA damage (Zheng et al., 2017; Zhou et al., 2017). Compared to the adult brain, the neonatal brain has a high level of mitochondrial respiration for oxygen consumption and low concentrations of antioxidants, which makes it particularly sensitive to the devastating consequences of oxidative stress (Bhat et al., 2015; Wu et al., 2019). Accumulating evidence has suggested that oxidative stress may contribute to sevoflurane-induced neurotoxicity in the developing brain (Yonamine et al., 2013; Liu et al., 2019). In a neonatal rat model, sevoflurane exposure could induce intracellular ROS production through associating with mitochondrial dysfunction and activation of NADPH oxidase, which results in widespread neurodegeneration and long-term behavioral impairment (Yonamine et al., 2013; Sun et al., 2016). Our results found both *in vitro* and *in vivo* that sevoflurane exposure was associated with increased accumulation of intracellular ROS. In contrast, pretreatment with NAC significantly prevented sevoflurane-induced overproduction of ROS, DNA strand nicks and breaks, and elevation of γH2AX, p-ATM, and 8-OHdG, which is a marker of oxidative DNA base damage, indicating that ROS contributed to sevoflurane-induced DNA damage in the developing brain. Oxidative stress-induced DNA damage differentially triggered the activation of PARP-1 and, in consequence, provoked different cell fate decisions. After mild oxidative DNA damage, PARP-1 activation exerts to engage in DNA repair and cell survival, whereas upon severe DNA damage due to massive oxidative stress, PARP-1 overactivation drives cell death response (Ray Chaudhuri and Nussenzweig, 2017). Chiu et al. (2011) reported that enhanced DNA damage caused by excess ROS accumulation triggered PARP-1-dependent cell death in mouse embryonic fibroblasts. Wang et al. (2018) also proved that oxidative DNA damage was associated with cell death triggered by PARP-1 overactivation. Consistently, our results in this study showed that sevoflurane-induced neuronal cell death and upregulation of PARP-1, PAR polymer, and nuclear AIF were markedly suppressed when DNA damage was alleviated by ROS inhibitor NAC. In support, we demonstrated previously that antioxidant NAC prevented PARP-1 hyperactivation and neuronal Parthanatos in SH-SY5Y cells under oxygen-glucose deprivation (Wang et al., 2018). These findings threw light on the role of oxidative stress due to ROS accumulation by acting as an up-stream mechanism on DNA damage in sevoflurane-triggered neuronal cell Parthanatos. To further verify the role of ROS-initiated DNA damage in Parthanatos of neuronal cells, we used exogenous H_2_O_2_ to mimic the oxidative stress process in SH-SY5Y cells. We observed that H_2_O_2_ induced a significant increase in cell death and upregulation of PARP-1, PAR polymer, and nuclear AIF in SH-SY5Y cells, which were all mitigated in the presence of 3AB. In addition, pretreatment with NAC significantly suppressed H_2_O_2_-induced overproduction of ROS, upregulation of 8-OHdG, γH2AX, and p-ATM, and expression of Parthanatos-related proteins, as well as cell death in SH-SY5Y cells. Taken together, these results suggested that ROS-initiated DNA damage contributed to Parthanatos of neuronal cells.

General anesthetic exposure during the developmental stages of the brain can cause persistent cognitive dysfunction in various mammalian species (Amrock et al., 2015; Liu et al., 2015; Diaz et al., 2016; Alvarado et al., 2017). It has been reported that neuronal apoptosis and impaired synaptogenesis are considered significant processes associated with cognitive impairment in neonatal animals (Kodama et al., 2011; Yang et al., 2011; Mintz et al., 2013; Olutoye et al., 2016; Sun et al., 2019). The present study also demonstrated that PARP-1 inhibitor has a potential therapeutic effect on memory deficits induced by neonatal sevoflurane exposure. We found that PARP-1 inhibitor 3AB or antioxidant NAC not only inhibited hippocampal neuronal death, but also rescued spatial memory deficits in neonatal rats following sevoflurane exposure, suggesting that PARP-1-dependent cell death participated in cognitive impairment induced by neonatal sevoflurane exposure. This result is consistent with a recent finding that the treatment of PJ34 (another PAPR-1 inhibitor) improved impaired memory and neuronal deficits after cerebral ischemic injury (Kauppinen et al., 2009; Haddad et al., 2013; El Amki et al., 2018).

While this study has provided some interesting data, it also has limitations. Although our findings suggest that sevoflurane induced oxidative stress and resultant DNA damage, which resulted in PARP-1-depentdent cell death and cognitive dysfunction, the effect of oxidative stress-induced inflammation on neurodegeneration was not considered in the present study. Previous studies have proven that sevoflurane-induced excessive ROS generation activated the microglia and amplified the production of inflammatory cytokines including IL-1β, IL-6, and TNF-α, linking to the occurrence of neurodegeneration (Shen et al., 2013; Su et al., 2019; Wu et al., 2020). It is also reported that PARP-1 hyperactivation was associated with the activation of high-mobility group box-1 (HMGB1) inflammatory signal pathway, which resulted in neuroinflammation and neurodegeneration (Qin et al., 2015; Tajuddin et al., 2018). However, our pilot study and data from other studies fall short of providing a direct link between inflammation and Parthanatos. Thus, future studies are considered to investigate this potential phenomenon and to further elucidate the mechanism of anesthetic-induced developmental neurotoxicity.

## Conclusion

In summary, our data first evidenced that Parthanatos contributed to neonatal neuronal cell death induced by sevoflurane exposure, which was initiated by oxidative DNA damage via the increase of intracellular ROS. Our findings provide critical insights into the mechanisms of sevoflurane-induced developmental neurotoxicity and highlight a potential therapeutic target for preventing neuronal injury from Parthanatos that contributes to the volatile anesthetics-induced neuronal cell death when anesthesia is administered to infants and children.

## Data Availability Statement

The original contributions presented in the study are included in the article/Supplementary Material, further inquiries can be directed to the corresponding author/s.

## Ethics Statement

The animal study was reviewed and approved by the Ethics Committee of The First Hospital of Jilin University.

## Author Contributions

MP: writing – original draft and funding acquisition. YW: investigation and validation. NL: software, validation, formal analysis, and data curation. XW: investigation. RC: visualization and investigation. JQ: formal analysis and investigation. PG: validation and data curation. CF: conceptualization, methodology, supervision, funding acquisition, writing, review, and editing. All authors contributed to the article and approved the submitted version.

## Conflict of Interest

The authors declare that the research was conducted in the absence of any commercial or financial relationships that could be construed as a potential conflict of interest.

## Abbreviations

AIF: apoptosis-inducing factor
ATM: ataxia telangiectasia mutated
ATP: adenosine triphosphate
8-OHdG: 8-hydroxydeoxyguanosine
BSA: bovine serum albumin
DCFH-DA: dichloro-dihydro-fluorescein diacetate
DMEM: Dulbecco’s modified eagle medium
DNA: deoxyribonucleic acid
DSBs: double strand breaks
EB: ethidium bromide
ELISA: enzyme-linked immunosorbent assay
FBS: fetal bovine serum
γ H2AX: phosphorylation of histone variant H2AX at Ser139
HE: hematoxylin and eosin
H_2_O_2_: hydrogen peroxide
HMGB1: high-mobility group box-1
i.p.: intraperitoneally
LDH: lactate dehydrogenase
MTT: methyl thiazolyl tetrazolium
NAC: *N*-acetyl -L-cysteine
NAD: nicotinamide adenine dinucleotide
PAR: polymerized ADP-ribose
PARP-1: poly (ADP-ribose) polymerase 1
PBS: phosphate buffered saline
P7: postnatal day 7
PVDF: polyvinylidene difluoride
ROS: reactive oxygen species
3AB: 3-aminobenzamide
SCGE: single cell gel electrophoresis
SD: Sprague-Dawley
SDS-PAGE: sodium dodecyl sulfate-polyacrylamide gel electrophoresis
SiRNA: small interfering RNA
SSBs: single strand breaks.
