# Supplementary material for: Sevoflurane Exposure Induces Neuronal Cell Parthanatos Initiated by DNA Damage in the Developing Brain via an Increase of Intracellular Reactive Oxygen Species
Source: Front Cell Neurosci. 2020 Dec 3;14:583782. doi: 10.3389/fncel.2020.583782 (PMC7793874; doi:10.3389/fncel.2020.583782)
Supplement: Supplementary file 1 [file Data_Sheet_1.docx]

**Supplementary Figure and Legend**

**
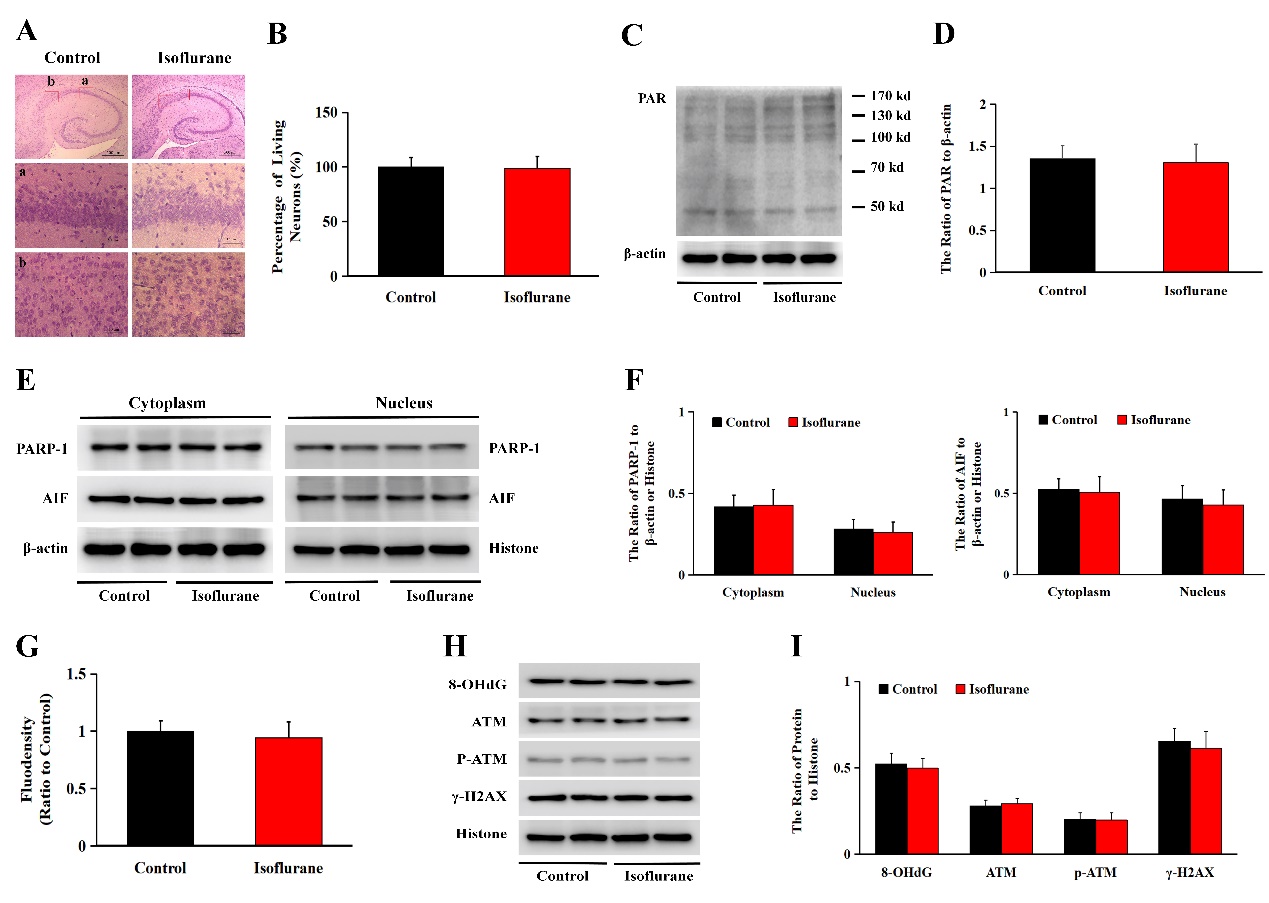
**

**Euthanasia with 5% isoflurane for 6 min showed no neurotoxicity in hippocampus of neonatal rats in our pilot study.** Twelve P7 rats from one litter including both male and female were randomly divided into two groups (n=6 per group): Control group, rats were sacrificed by cervical dislocation; Isoflurane group, rats were euthanized with 5% isoflurane for 6 min. **(A, B)** Representative images of hippocampal neurons stained by hematoxylin and eosin (HE) staining in P7 rat pups after 5% isoflurane exposure for 6 min. Scale bar=50 μm. Compared with the control group, 5% isoflurane exposure for 6 min showed no significant differences in the number, arrangement, or morphological changes of the pyramidal neurons in hippocampal CA1 region. **(C-F)** Western blotting and quantitative analysis showed that no significant differences were found in the levels of PAR, PARP-1 and nuclear AIF in hippocampus of rat pups after 5% isoflurane exposure for 6 min, when compared to the control group. **(G)** ELISA analysis showed that there were no significant changes in the levels of ROS in hippocampus of rat pups after 5% isoflurane exposure for 6 min, when compared with the control group. **(H, I)** Western blotting and quantitative analysis showed that no significant differences were found in the levels of 8-OHdG, γH2AX and p-ATM in hippocampus of rat pups after 5% isoflurane exposure for 6 min, when compared to the control group. Statistical differences were analyzed using a two-tailed unpaired *t*-test. Experimental data were expressed as mean ± SD.
